# Supplementary material for: Polyketide synthase gene clusters in the ericoid mycorrhizal fungus Oidiodendron maius: genome-wide prediction, expression profiling, and functional characterization of knockout mutants
Source: Front Plant Sci. 2026 Sep 16;17:1924430. doi: 10.3389/fpls.2026.1924430 (PMC13623903; doi:10.3389/fpls.2026.1924430)
Supplement: Supplementary file 1 [file SupplementaryFile1.zip › Supplementary Materials/Supplementary Folder 1.zip!/Oidma1_AssemblyScaffolds_Repeatmasked/knownclusterblast/cluster48/jgi.p_Oidma1_136654_mibig_hits.html]

| MIBiG Protein | Description | MIBiG Cluster | MiBiG Product | % ID | % Coverage | BLAST Score | E-value |
| --- | --- | --- | --- | --- | --- | --- | --- |
| EPE34350.1 | Clavaminate\_synthase-like\_protein | BGC0001035 | Pneumocandin\_biosynthetic\_gene\_cluster | 39 | 102.102102102 | 231 | 1.7e-60 |
| AGA37270.1 | 2OG-Fe(II)-oxygenase | BGC0000819 | Paraherquamide\_biosynthetic\_gene\_cluster | 39 | 103.303303303 | 224 | 2.1e-58 |
| AFT91391.1 | HtyE | BGC0000338\_c2 | Echinocandin\_B\_biosynthetic\_gene\_cluster | 36 | 101.501501502 | 200 | 3.2e-51 |
| AMM63174.1 | AniF | BGC0001371 | Echinocandin\_B\_biosynthetic\_gene\_cluster | 36 | 101.501501502 | 200 | 4.2e-51 |
| EPE34347.1 | Clavaminate\_synthase-like\_protein | BGC0001035 | Pneumocandin\_biosynthetic\_gene\_cluster | 36 | 101.801801802 | 189 | 5.7e-48 |
| Solyc07g043420.2 |  | BGC0001319 | alpha-Tomatine\_biosynthetic\_gene\_cluster. | 27 | 79.2792792793 | 97 | 2.2e-20 |
| PGSC0003DMG400011751 |  | BGC0001320 | alpha-Solanine\_/\_alpha-Chaconine\_biosynthetic\_gene\_cluster. | 26 | 79.2792792793 | 91 | 2.1e-18 |
| CAM59607.1 | putative\_dioxygenase | BGC0000297 | Aeruginoside\_biosynthetic\_gene\_cluster | 30 | 61.5615615616 | 89 | 8e-18 |
| EED18007.1 | 1-aminocyclopropane-1-carboxylate\_oxidase,\_putative | BGC0000154 | Stipitatic\_acid\_biosynthetic\_gene\_cluster | 26 | 93.0930930931 | 82 | 7.5e-16 |
